# Catalytic Annulation of Epoxides with Heterocumulenes by the Indium-Tin System

**DOI:** 10.3390/molecules23040782

**Published:** 2018-03-28

**Authors:** Itaru Suzuki, Akira Imakuni, Akio Baba, Ikuya Shibata

**Affiliations:** Research Center for Environmental Preservation, Osaka University, Osaka 565-0871, Japan; suzuki@epc.osaka-u.ac.jp (I.S.); imakuni@epc.osaka-u.ac.jp (A.I.); baba@chem.eng.osaka-u.ac.jp (A.B.)

**Keywords:** indium, tin, epoxide, carbon dioxide, isocyanate, cyclic carbonate, 2-oxazolidinone

## Abstract

In the synthesis of five-membered heterocycles by the annulation of epoxides with heterocumulenes such as carbon dioxide and isocyanates, we developed the indium-tin catalytic system and synthesized various cyclic adducts including novel types products under mild reaction conditions.

## 1. Introduction

Synthesis of five-membered heterocycles by the annulation of epoxides with heterocumulenes such as carbon dioxide and isocyanates has been intensively studied [1,2,3]. For the use of carbon dioxide, its catalytic transformation to useful organic compounds has attracted much attention [4,5,6,7,8]. In particular, fixation of carbon dioxide to cyclic carbonates is one of the most important processes of high atom efficiency [9]. Cyclic carbonates are known to be efficient aprotic polar solvents [10,11,12], electrolytes in lithium ion batteries [13,14] and materials for producing polycarbonates [15]. Instead of the products from carbon dioxide, the cyclic adducts of epoxides with isocyanates such as 2-oxazolidinones [16,17] are important biologically-active compounds [18,19,20,21,22] and synthetic intermediates such as precursors of amino alcohols and chiral auxiliaries [23,24,25,26,27,28,29,30,31]. We present here a simple, easily available catalyst, the indium-tin system, and provide the environmentally-benign process to annulated adducts under mild conditions.

## 2. Results

### 2.1. Synthesis of Cyclic Carbonates

Until now, various catalysts such as transition metal compounds, ionic liquid, onium salts and alkali metal salts, etc., have been developed for the reaction of epoxides with carbon dioxide [5,32,33]. However, these methods suffer from either the need for co-solvent, the requirement for high temperature, high CO_2_ pressure or expensive catalyst. Indium reagents and catalysts have been applied in modern organic synthesis by their mildness and easy handling character [34]. By using the indium halide-phosphine complex, we have already developed the reaction of terminal epoxides to give cyclic carbonates under atmospheric CO_2_ pressure at room temperature [35], which indicated that indium halide-based catalysts have efficient catalytic activities. As shown in Table 1, we screened indium halide catalytic systems in the reaction of epoxide **1a** with CO_2_ (3.9 MPa) at room temperature. The sole use of InCl_3_ did not have catalytic activity (Entry 1). Interestingly, the combination of Bu_2_SnI_2_ with InCl_3_ increased the yield of carbonate **2a** (Entry 2). The sole use of Bu_2_SnI_2_ was not effective at all (Entry 3). Thus, the InCl_3_-Bu_2_SnI_2_ system showed a high catalytic activity. Of particular interest is that the reaction proceeded well even at room temperature. The choice of acetonitrile as a solvent is essential because no reaction proceeded when other solvents such as hexane, benzene, CHCl_3_ and THF were used. In acetonitrile, the reaction proceeded very well, and various cyclic carbonates **2** were obtained from epoxides **1b**–**1f** catalyzed by InCl_3_-Bu_2_SnI_2_. Epoxides having aliphatic and aromatic substituents were reactive to afford the corresponding cyclic carbonates **2b**–**2c** (Entries 4 and 5). High chemoselectivities were observed because of the mild conditions. Functionalized cyclic carbonates **2d**–**2f** were synthesized from epoxides having halogen and oxygen substitutes (Entries 6–8). 

### 2.2. Synthesis of 2-Oxazolidinones

Instead of using carbon dioxide, the cyclic adducts of epoxides with isocyanates are highly in demand [18,19,20,21,22,23,24,25,26,27,28,29,30,31]. To effect the reaction, various catalysts have been used such as lithium halides [36,37,38,39,40,41,42], quaternary ammonium salts [43,44], phosphonium salt [45], AlCl_3_ [46], magnesium halides [47], tetraphenylantimony iodide [48,49,50] and the chromium(Salphen) complex [51]. These catalysts promoting reactions require relatively severe conditions (over 100 °C). The Pd-catalyzed reaction enabled mild conditions and accomplished an asymmetric reaction; however, in the reaction, only vinyl-substituted epoxides could be applicable where π-allyl palladium intermediates should be generated [52]. We have already reported that the Bu_3_SnI-Ph_3_PO or Ph_4_SbI catalyzes the annulation of epoxides with aromatic isocyanates to give 3,5-disubstututed-2-oxazolidinones [53,54,55,56], which indicated that tin halide-based catalysts would afford efficient catalytic activity. In view of these backgrounds, we tested the catalytic activity of the Bu_2_SnI_2_-InCl_3_ system as shown in Table 2. In the reaction of epoxybutane **1b** with *tert*-BuN=C=O (**3a**), a quantitative yield of 2-oxazolidinone **4a** was obtained (Entry 1). Interestingly, steric hindrance of isocyanates was not a problem to give 2-oxazolidinones **4**. The use of either Bu_2_SnI_2_ or InCl_3_ was not effective (Entries 2 and 3). Thus, it was clear that the InCl_3_-Bu_2_SnI_2_ catalytic system showed a high activity even for the synthesis of 2-oxazolidinones **4**. Other epoxides such as epichlorohydrin **1d** and glycidylic ethers **1e**, **1f** also reacted well (Entries 4–6). Of course, primary aliphatic isocyanate **3b** and phenyl isocyanate **3c** also gave the desired products **4e** and **4f**, although the yields were moderate owing to the trimerization of an isocyanate as a side reaction (Entries 7 and 8) [51,57]. Thus, higher yields of **4a**–**d** in the reaction of *tert*-BuN=C=O (**3a**) were achieved because the trimerization would be depressed by steric hindrance of **3a**. In all cases, regioselective ring opening of epoxides took place at the less substituted site to give 3,5-disubstituted-2-oxazolidinones **4**. 

**Scheme 1:**
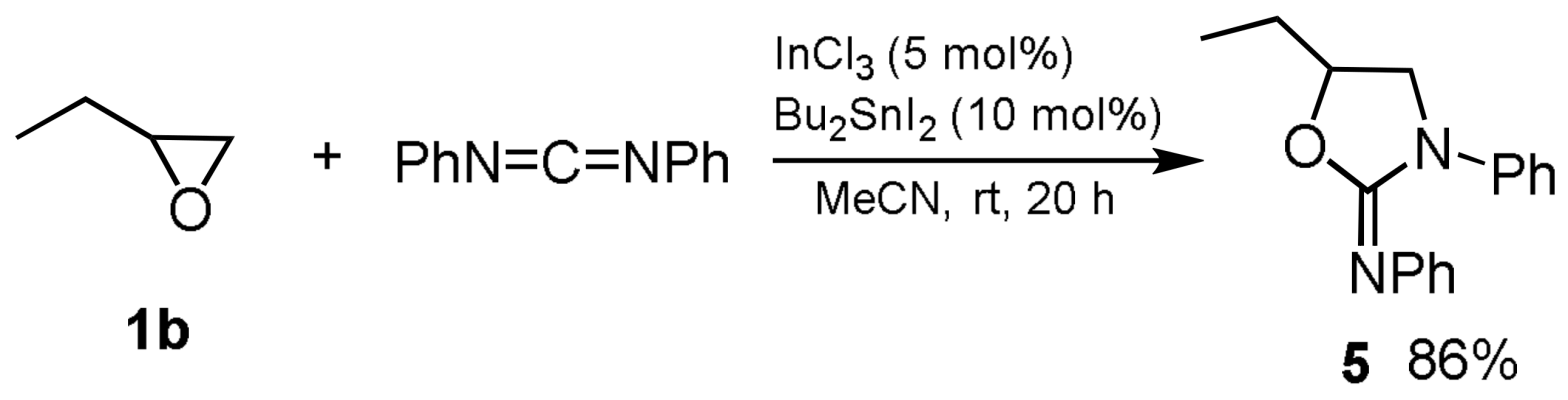
Catalytic synthesis of oxazolidin-2-imine **5**.

In the reaction with diphenyl carbodiimide, an analogue of isocyanates, the oxazolidin-2-imine **5**, was obtained in good yield (Scheme 1).

## 3. Discussion

**Figure 1:**
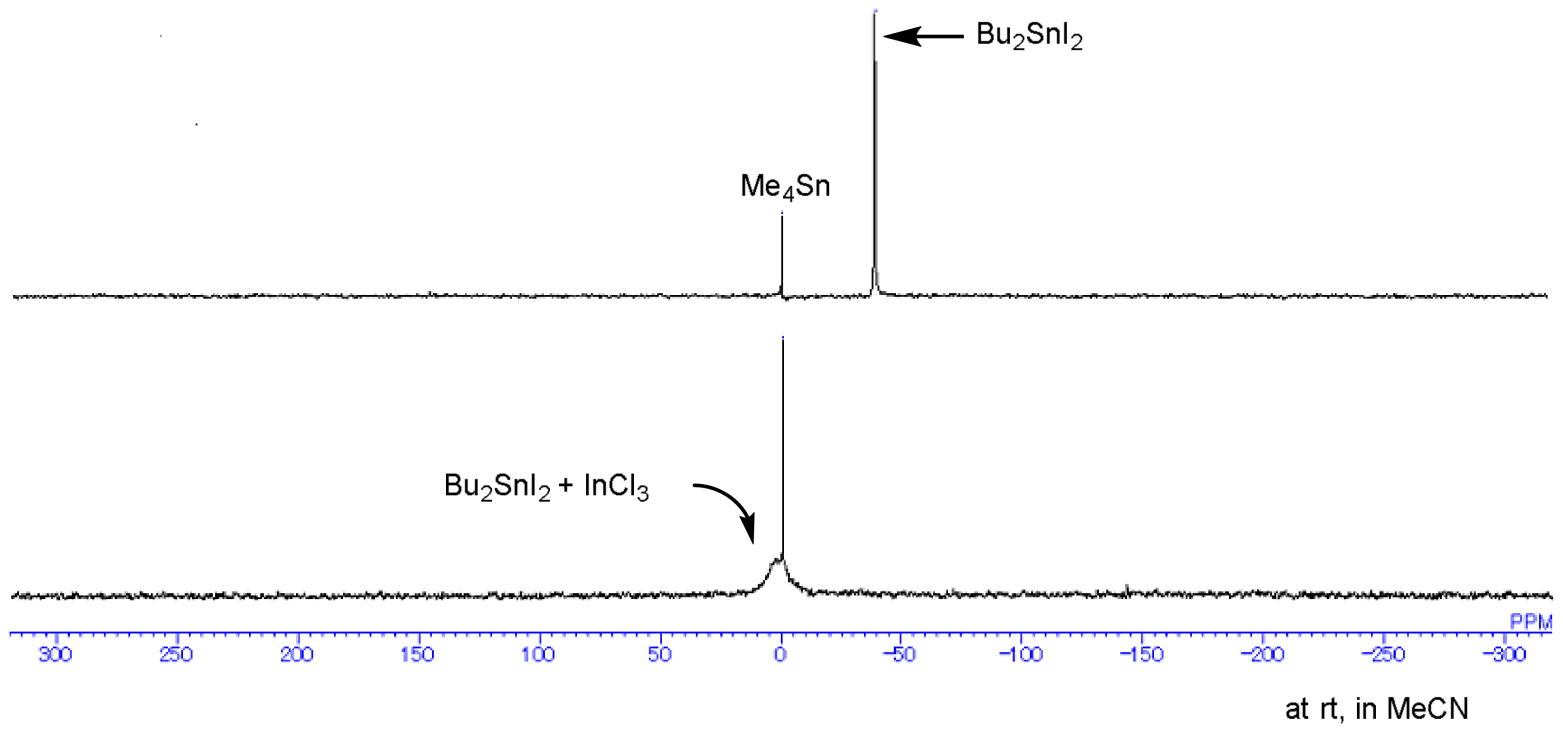
^119^Sn NMR of the tin-indium system.

As shown in Figure 1, the structure of the Bu_2_SnI_2_-InCl_3_ system could be supposed by the measurement of ^119^Sn-NMR spectra in acetonitrile. The addition of equimolar InCl_3_ to Bu_2_SnI_2_ in acetonitrile clearly changed the ^119^Sn peak from a strong one at −38 ppm to a broad one at 3 ppm. This downfield shift indicates that tin species had a positive character by the combination with InCl_3_ [58,59].

**Scheme 2:**
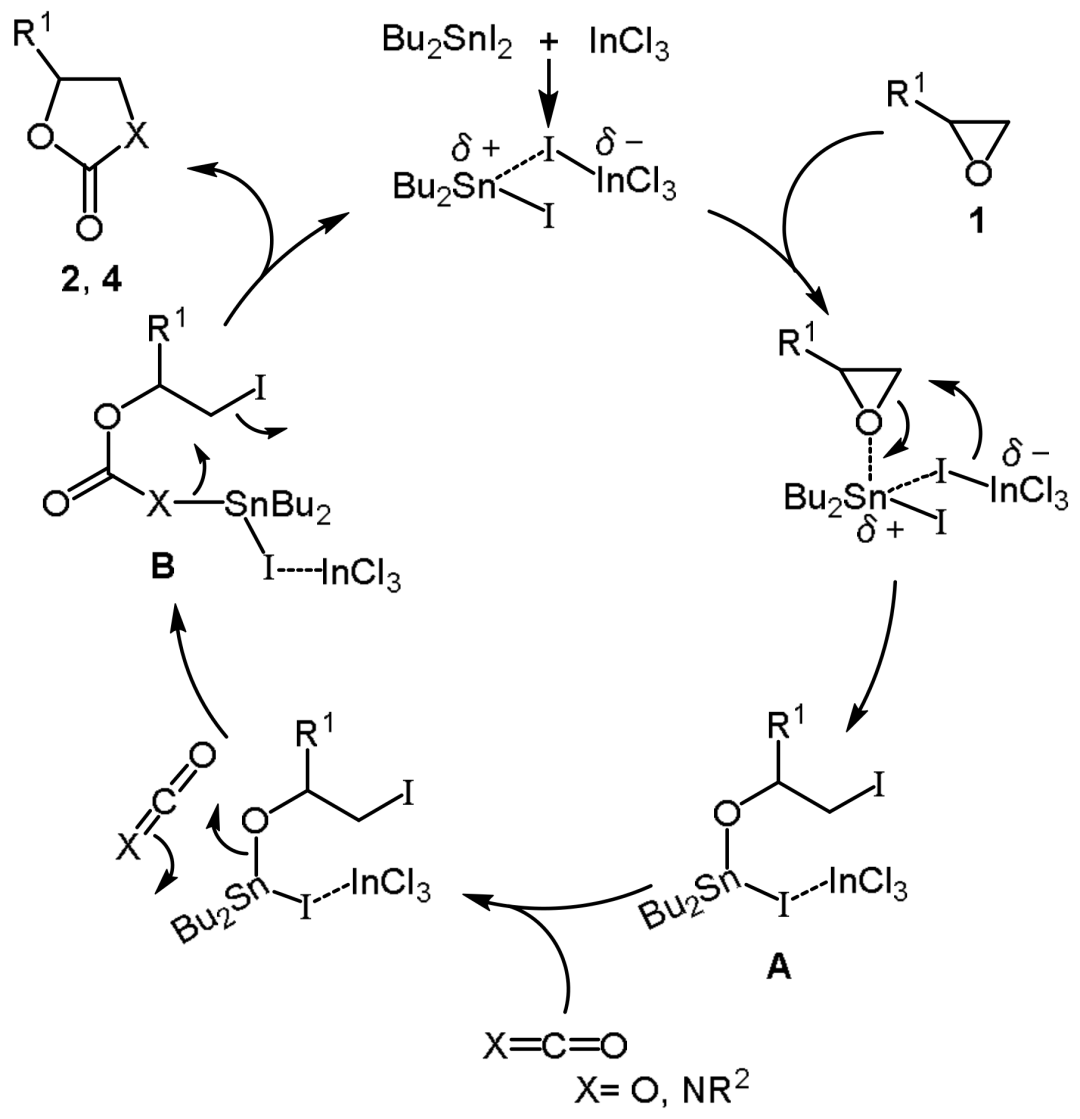
A plausible catalytic cycle.

The catalytic cycle is explained as shown in Scheme 2. By the interaction of tin and indium species, Lewis acidic tin species like Bu_2_SnI*^δ^*^+^[InCl_3_I]*^δ^*^−^ would be generated, which activate the epoxide ring [60,61,62,63]. This active bimetallic species is plausible because transmetallation between tin and indium reagents easily takes place [64,65,66,67,68]. The ring opening of an epoxide to **A** proceeds regioselectively at the less substituted carbon. Next, the tin-oxygen bond in **A** is added to heterocumulene to give an adduct **B**. In the case of isocyanates, the addition occurs at the C=N group selectively to give a stannylcarbamate **B** [69,70,71]. At the last stage, the Sn-X (X=O, NR) bond in the intermediate **B** attacks the terminal alkyl iodide [72,73] to afford cyclic carbonates **2** and 2-oxazolidinones **4** and regenerate the catalyst. The ^1^H-NMR of products **4** showed single regio isomers because of the regioselective ring opening of epoxides. For example, 5-H and 4-H peaks for **4f** were found at 4.59 (1H), 4.08–3.66 (2H) ppm, respectively. On the other hand, it has been reported by us that ^1^H-NMR for another regio isomer showed its 5-H and 4-H peaks at 4.65–4.30 (2H), 4.22–4.04 (1H) ppm, respectively [49].

## 4. Materials and Methods

### 4.1. Analysis

FTIR spectra were recorded as a thin film on a Nicolet IS5 spectrometer (Thermo Electron Scientific Instruments LLC, Madison, WI, USA). All ^1^H and ^13^C-NMR spectra were recorded with a JEOL JMTC-400/54/SS (400 and 100 MHz, respectively) in deuteriochloroform (CDCl_3_) containing 0.03% (*w*/*v*) of tetramethylsilane as an internal standard. Temperatures shown in schemes or tables were controlled by a constant-temperature oil bath. Yields were determined by ^1^H-NMR using 1,1,1,2-tetrachloroethane or 1,1,2,2-tetrachloroethane as an internal standard. Mass spectra were recorded on a JEOL JMS-DS-303 spectrometer (JEOL Ltd., Tokyo, Japan). Flash column chromatography was performed by Yamazen YFLC-AI-580 using Hi-Flash Silica gel 2L Hi-Flash Column 20 mL/min eluted by hexane/EtOAc with the gradation mode changing from 9/1–3/7 depending on R_f_ values of each compound. Bulb-to-bulb distillation (Kugelrohr) was accomplished at the oven temperature and pressure indicated.

Dehydrated acetonitrile (MeCN) was purchased from commercial sources and used as obtained. Deuterated acetonitrile was also purchased and stored drying over 4 Å molecular sieves. All epoxides, isocyanates, carbodiimide and InCl_3_ were also purchased and used as obtained. Bu_2_SnI_2_ was prepared according to the previous report [74].

### 4.2. General Procedure for Synthesis of Cyclic Carbonates ***2a***–***f*** from Epoxides ***1*** with CO_2_

To a 50-mL autoclave, InCl_3_ (0.5 mmol), Bu_2_SnI_2_ (1.0 mmol) and epoxide **1** (10 mmol) were added in MeCN (3 mL). The autoclave was flushed with CO_2_ (3.9 MPa) and stirred at room temperature for 5–10 h. After release of the CO_2_ gas, the reaction mixture was quenched with H_2_O (20 mL) and extracted with Et_2_O (3 × 20 mL). The collected organic layer was dried over MgSO_4_. After filtration, the mixture was concentrated in vacuo. The residue was purified by column chromatography. Further purification was performed by Kugelrohr distillation to give a pure product **2**. 

*4-Methyl-1.3-dioxolan-2-one* (**2a**). Colorless liquid. The NMR data of ^1^H and ^13^C agreed with the previous report. [75]. ^1^H-NMR: (270 MHz, CDCl_3_) *δ* 4.70 (ddd, *J* = 6.4, 7.3, 7.8 Hz, 1H, 4-H), 4.38 (dd, *J* = 7.8, 8.3 Hz, 1H, 5*H*H), 3.84 (dd, *J* = 7.3, 8.3 Hz, 1H, 5H*H*), 1.29 (d, *J* = 6.34 Hz, 3H, 4-C*H*_3_). ^13^C-NMR: (67.5 MHz, CDCl_3_) *δ* 155.00 (C-2), 73.55 (C-4), 70.58 (C-5), 19.18 (4-*C*H_3_).

*4-Ethyl-1.3-dioxolan-2-one* (**2b**). Colorless liquid. The NMR data of ^1^H and ^13^C agreed with the previous report [76]. ^1^H-NMR: (270 MHz, CDCl_3_) *δ* 4.70–4.65 (m, 1H, 4-H), 4.56 (dd, *J* = 8.3, 8.3 Hz, 1H, 5*H*H), 4.12 (dd, *J* = 7.8, 7.8 Hz, 1H, 5H*H*) 1.88–1.68 (m, 2H, 4-C*H*_2_CH_3_), 1.03 (t, *J* = 7.3 Hz, 3H, 4-CH_2_C*H*_3_). ^13^C NMR: (67.5 MHz, CDCl_3_) *δ* 154.91 (C-2), 77.88 (C-4), 68.83 (C-5), 26.59 (4-*C*H_2_CH_3_), 8.20 (4-CH_2_*C*H_3_).

*4-Phenyl-1.3-dioxolan-2-one* (**2c**). Colorless liquid. The NMR data of ^1^H and ^13^C agreed with the previous report [75]. ^1^H-NMR: (270 MHz, CDCl_3_) *δ* 7.44–7.18 (m, 5H, Ph), 5.66 (t, *J* = 8.1 Hz, 1H, 4-H) 4.78 (dd, *J* = 8.3, 8.3 Hz, 1H, 5*H*H), 4.33 (dd, *J* = 7.8, 7.8 Hz, 1H, 5H*H*). ^13^C-NMR: (67.5 MHz, CDCl_3_) *δ* 154.71 (C-2), 135.67 (*i*), 129.62 (*m*), 129.12 (*p*), 125.77 (*o*), 77.92 (C-4), 71.10 (C-5).

*4-(Chloromethyl)-1,3-dioxolan-2-one* (**2d**). Colorless liquid. The NMR data of ^1^H and ^13^C agreed with the previous report [75]. ^1^H-NMR: (270 MHz, CDCl_3_) *δ* 5.10–5.02 (m, 1H, 4-H), 4.63 (dd, *J* = 8.8, 8.8 Hz, 1H, 5*H*H), 4.41 (dd, *J* = 5.8, 8.8 Hz, 1H, 5H*H*), 3.88 (dd, *J* = 4.4, 12.2 Hz, 1H, C*H*HCl), 3.76 (dd, *J* = 3.4, 12.2 Hz, 1H, CH*H*Cl). ^13^C-NMR: (67.5 MHz, CDCl_3_) *δ* 154.33 (C-2), 74.33 (C-4), 66.70 (C-5), 44.01 (*C*H_2_Cl).

*4-(Phenoxymethyl)-1,3-dioxolan-2-one* (**2e**). Colorless liquid. The NMR data of ^1^H and ^13^C agreed with the previous report [76]. ^1^H-NMR: (270 MHz, CDCl_3_) *δ* 7.35–6.87 (m, 5H, Ph), 5.08–4.99 (m, 1H, 4-H), 4.62 (dd, *J* = 8.3, 8.8 Hz, 1H, C*H*HOPh), 4.54 (dd, *J* = 6.4, 8.8 Hz, 1H, CH*H*OPh), 4.25 (dd, *J* = 3.9, 10.3 Hz, 1H, 5H*H*), 4.15 (dd, *J* = 3.9, 10.3 Hz, 1H, 5*H*H). ^13^C-NMR: (67.5 MHz, CDCl_3_) *δ* 157.72 (*o*), 154.67 (C-2), 129.55 (*p*), 121.98 (*m*), 114.59 (*m*), 74.10 (*C*H_2_OPh), 66.87 (C-4), 66.23 (C-5).

*4-(Methoxymethyl)-1,3-dioxolan-2-one* (**2f**). Colorless liquid. The NMR data of ^1^H and ^13^C agreed with the previous report [35]. ^1^H-NMR: (270 MHz, CDCl_3_) *δ* 4.88–4.80 (m, 1H, 4-H), 4.51 (dd, *J* = 8.3, 8.8 Hz, 1H, 5*H*H), 4.37 (dd, *J* = 6.3, 8.3 Hz, 1H, 5H*H*), 3.67 (dd, *J* = 3.4, 11.2 Hz, 1H, C*H*HOCH_3_), 3.54 (dd, *J* = 3.9, 11.2 Hz, 1H, CH*H*OCH_3_), 3.43 (s, 3H, OC*H*_3_). ^13^C-NMR: (67.5 MHz, CDCl_3_) *δ* 154.91 (C-2), 75.01 (C-4), 71.27 (*C*H_2_OCH_3_), 65.97 (C-5), 59.34 (O*C*H_3_).

### 4.3. General Procedure for Synthesis of 2-Oxazolidinones ***4*** from Epoxides ***1b***–***f*** with Isocyanates ***3*** and Oxazolidin-2-imine ***5***

To a two-neck 10-mL reaction vessel, InCl_3_ (0.25 mmol), Bu_2_SnI_2_ (0.50 mmol), epoxide **1** (5 mmol) and isocyanate **3** (5.5 mmol) were added in MeCN (1.5 mL) under N_2_ atmosphere. The reaction mixture was stirred at room temperature or 60 °C for 3–20 h. After completion of the reaction, the mixture was quenched with H_2_O (20 mL) and extracted with Et_2_O (3 × 20 mL). The collected organic layer was dried over MgSO_4_. After filtration, the mixture was concentrated in vacuo. The residue was purified by column chromatography. For some cases, further purification was performed by distillation to give a pure product **4**.

*3-(tert-Butyl)-5-ethyloxazolidin-2-one* (**4a**). Colorless liquid. bp: 78 °C/2 mmHg. IR (neat): 1743.33 cm^−1^. HRMS: (EI+, 70 eV) Calculated (C_9_H_17_NO_2_) 171.1259 (M^+^) Found: 171.1257. ^1^H-NMR: (270 MHz, CDCl_3_) *δ* 4.30 (ddd, *J* = 6.4, 7.8, 8.8 Hz, 1H, 5-H), 3.64 (dd, *J* = 8.3, 8.8 Hz, 1H, 4*H*H), 3.20 (dd, *J* = 7.8, 8.3 Hz, 1H, 4H*H*), 1.70 (m, 2H, C*H*_2_CH_3_), 1.38 (s, 9H, NC(C*H*_3_)_3_), 0.99 (t, *J* = 7.3 Hz, 3H, CH_2_C*H*_3_), ^13^C-NMR: (67.5 MHz, CDCl_3_) *δ* 159.76 (C-2), 73.21 (C-5), 52.92 (N*C*(CH_3_)_3_), 48.06 (4-C), 27.45 (*C*H_2_CH_3_), 27.11 (NC(*C*H_3_)_3_), 8.55 (CH_2_*C*H_3_).

*3-(tert-Butyl)-5-(chloromethyl)oxazolidin-2-one* (**4b**). Colorless liquid. bp: 80 °C/2 mmHg. IR (neat): 1743.33 cm^−1^. HRMS: (EI+, 70 eV) Calculated (C_8_H_14_ClNO_2_) 191.0713 (M^+^) Found: 191.0714. ^1^H-NMR: (270 MHz, CDCl_3_) *δ* 4.64–4.55 (m, 1H, 5-H), 3.74 (dd, *J* = 8.8, 8.8 Hz, 1H, 4*H*H), 3.65 (dd, *J* = 5.9, 6.4 Hz, 2H, C*H*_2_Cl), 3.52 (dd, *J* = 5.9, 8.8 Hz, 1H, 4H*H*), 1.40 (s, 9H, NC(C*H*_3_)_3_). ^13^C-NMR: (67.5 MHz, CDCl_3_) *δ* 155.62 (C-2), 70.09 (C-5), 53.52 (N*C*(CH_3_)_3_), 46.00 (*C*H_2_Cl), 44.70 (C-4), 27.16 (NC(*C*H_3_)_3_).

*3-(tert-Butyl)-5-(phenoxymethyl)oxazolidin-2-one* (**4c**). Colorless liquid. bp: 140 °C/2 mmHg. IR (neat): 1727.91 cm^−1^. HRMS: (EI+, 70 eV) Calculated (C_9_H_17_NO_2_) 249.1365 (M^+^) Found: 249.1371. ^1^H-NMR: (270 MHz, CDCl_3_) *δ* 7.10 (m, 5H, Ph), 4.72 (m, 1H, 5-H), 4.10 (d, *J* = 2.9 Hz, 2H, OC*H*_2_), 3.76 (dd, *J* = 8.8, 8.8 Hz, 1H, 4*H*H), 3.59 (dd, *J* = 5.9, 8.8 Hz, 1H, 4H*H*), 1.40 (s, 9H, NC(C*H*_3_)_3_). ^13^C-NMR: (67.5 MHz, CDCl_3_) *δ* 158.09 (C-2), 156.24, 129.53, 121.45, 114.14, 69.57 (O*C*H_2_), 67.97 (C-5), 53.45 (N*C*(CH_3_)_3_), 45.55 (4-C), 27.36 (NC(*C*H_3_)_3_).

*3-(tert-Butyl)-5-(methoxymethyl)oxazolidin-2-one* (**4d**). Colorless liquid. bp: 75 °C/0.07 mmHg. IR (neat): 1743.33 cm^−1^. HRMS: (EI+, 70 eV) Calculated (C_9_H_17_NO_3_) 187.1208 (M^+^) Found: 187.1212. ^1^H-NMR: (270 MHz, CDCl_3_) *δ* 4.51 (m, 1H, 5-H), 3.64 (dd, *J* = 8.3, 8.8 Hz, 1H, 4*H*H), 3.52 (dd, *J* = 4.9 Hz, 2H, C*H*_2_O), 3.43 (dd, *J* = 6.4, 8.3 Hz, 1H, 4H*H*), 3.40 (s, 3H, OC*H*_3_), 1.38 (s, 9H, NC(C*H*_3_)_3_). ^13^C-NMR: (67.5 MHz, CDCl_3_) *δ* 156.30 (C-2), 72.66 (O*C*H_2_), 70.38 (C-5), 59.92 (O*C*H_3_), 53.13 (N*C*(CH_3_)_3_), 45.14 (4-C), 27.14 (NC(*C*H_3_)_3_).

*3-Butyl-5-ethyloxazolidin-2-one* (**4e**). Colorless liquid. bp: 85 °C/2 mmHg. IR (neat): 1751.05 cm^−1^. HRMS: (EI+, 70 eV) Calculated (C_9_H_17_NO_2_) 171.1259 (M^+^) Found: 171.1257. ^1^H-NMR: (270 MHz, CDCl_3_) *δ* 4.72 (m, 1H, 5-H), 3.59 (dd, *J* = 8.3, 8.3 Hz, 1H, 4*H*H), 3.25 (m, 2H, NC*H*_2_), 3.14 (dd, *J* = 6.8, 8.3 Hz, 1H, 4H*H*), 1.83–1.64 (m, 2H, NCH_2_C*H*_2_), 1.53 (dt, *J* = 7.3, 7.3 Hz, 2H, C*H*_2_CH_3_), 1.34 (tq, *J* = 7.3, 7.3 Hz, 2H, NCH_2_CH_2_C*H*_2_), 1.00 (t, *J* = 7.3 Hz, 3H, CH_2_C*H*_3_), 0.94 (t, *J* = 7.3 Hz, 3H, NCH_2_CH_2_CH_2_C*H*_3_). ^13^C-NMR: (67.5 MHz, CDCl_3_) *δ* 157.81 (C-2), 74.18 (C-5), 49.06 (C-4), 43.39 (N*C*H_2_), 29.02 (NCH_2_*C*H_2_), 27.70 (*C*H_2_CH_3_), 19.46 (NCH_2_CH_2_*C*H_2_), 13.34 (NCH_2_CH_2_CH_2_*C*H_3_), 8.39 (CH_2_*C*H_3_).

*5-Ethyl-3-phenyloxazolidin-2-one* (**4f**). Colorless liquid. The NMR data of ^1^H and ^13^C agreed with the previous report [77]. ^1^H-NMR: (270 MHz, CDCl_3_) *δ* 7.56–7.10 (m, 5H, Ph), 4.59 (ddt, *J* = 6.4, 7.3, 8.3 Hz, 1H, 5-H), 4.08 (dd, *J* = 8.3, 8.8 Hz, 1H, 4*H*H), 3.66 (dd, *J* = 7.3, 8.8 Hz, 1H, 4H*H*), 1.96–1.71 (dq, *J* = 6.4, 7.3 Hz, 2H, C*H*_2_CH_3_), 1.07 (t, *J* = 7.3 Hz, 3H, CH_2_C*H*_3_). ^13^C-NMR: (67.5 MHz, CDCl_3_) *δ* 154.88 (C-2), 138.29, 128.91, 123.79, 118.06, 74.05 (C-5), 49.94 (C-4), 27.88 (*C*H_2_CH_3_), 8.62 (CH_2_*C*H_3_).

*5-Ethyl-N,3-diphenyloxazolidin-2-imine* (**5**). Colorless liquid. The NMR data of ^1^H and ^13^C agreed with the previous report [56]. ^1^H-NMR: (270 MHz, CDCl_3_) *δ* 8.23–6.60 (m, 10H, Ph × 2), 4.70–4.15 (m, 1H, 5-H), 3.92 (t, *J* = 8.1 Hz, 4-*H*H), 3.52 (dd, *J* = 6.8, 8.1 Hz, 1H, 4H*H*), 1.95–1.35 (m, 2H, C*H*_2_CH_3_), 0.97 (t, *J* = 6.8 Hz, 3H, CH_2_C*H*_3_). ^13^C-NMR: (67.5 MHz, CDCl_3_) *δ* 148.79, 148.05, 140.44, 139.34, 129.03, 124.03, 123.30, 122.82, 119.34, 76.16 (C-5), 50.74 (C-4), 27.44 (*C*H_2_CH_3_), 8.78 (CH_2_*C*H_3_).

### 4.4. Observation of Tin-Indium System by ^119^Sn NMR

To a two-neck 10-mL reaction vessel, InCl_3_ (1.0 mmol) and Bu_2_SnI_2_ (1.0 mmol) were added in MeCN-*d*_3_ (1 mL) and stirred at room temperature for several minutes. After transferring of the reaction mixture into an NMR test tube and addition of tetramethyl stannane as an internal standard, ^119^Sn NMR was recorded.

## 5. Conclusions

A novel type of indium-tin species Bu_2_SnI*^δ^*^+^[InCl_3_I]*^δ^*^−^ was revealed to be effective for the catalytic annulation of epoxides. In the reaction with carbon dioxide, the fixation of CO_2_ proceeded well even at room temperature. In the reaction with isocyanates, novel types of 2-oxazolidiones were obtained in good yields.

## Figures and Tables

**Table 1:**
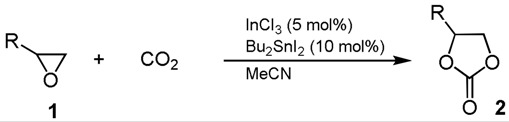
Synthesis of cyclic carbonates **2** from epoxides **1** with CO_2_ at rt ^a^.

Entry	R	Cat.	Time (h)	Product		Yield 2 (%) ^b^
1	Me (**1a**)	InCl_3_	5	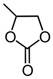	**2a**	trace
2		InCl_3_-Bu_2_SnI_2_	5			78
3		Bu_2_SnI_2_	5			trace
4	Et (**1b**)	InCl_3_-Bu_2_SnI_2_	8	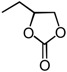	**2b**	85
5	Ph (**1c**)	InCl_3_-Bu_2_SnI_2_	10	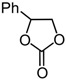	**2c**	69
6	CH_2_Cl (**1d**)	InCl_3_-Bu_2_SnI_2_	5	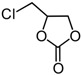	**2d**	82
7	CH_2_OPh (**1e**)	InCl_3_-Bu_2_SnI_2_	5	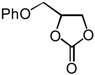	**2e**	90
8	CH_2_OMe (**1f**)	InCl_3_-Bu_2_SnI_2_	10	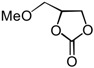	**2f**	68

^a^ InCl_3_ 0.5 mmol, Bu_2_SnI_2_ 1 mmol, epoxide **1** 10 mmol, CO_2_ 3.9 Pa, MeCN 3 mL; ^b^ Determined by ^1^H-NMR.

**Table 2:**
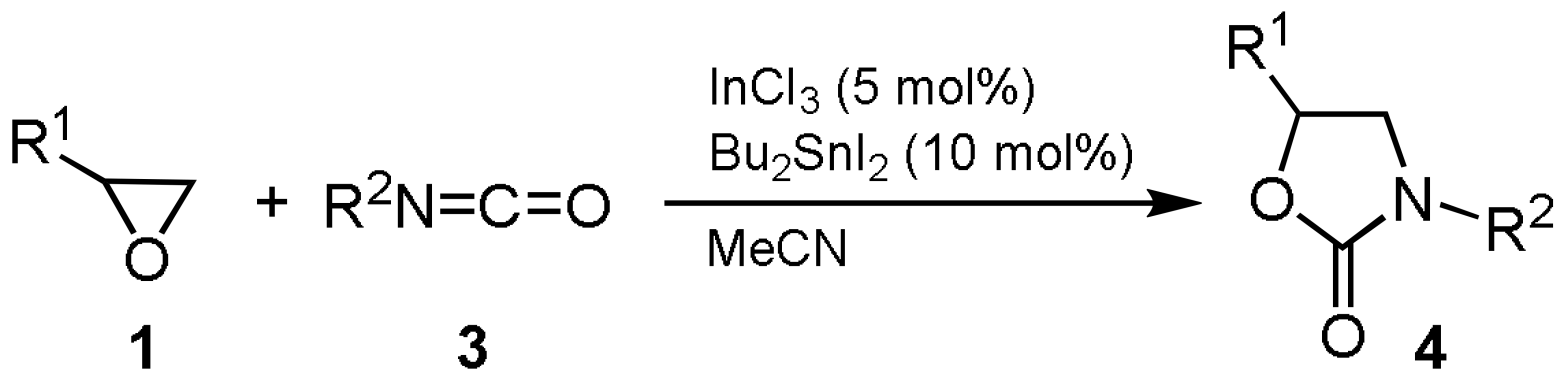
Synthesis of 2-oxazolidinones **4** from epoxides **1b**–**f** with isocyanates **3** ^a^.

Entry	R^1^	R^2^	Conditions	Product		Yield 3 (%) ^b^
1	Et (**1b**)	*t*-Bu (**3a**)	rt, 10 h	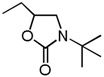	**4a**	79
2						7 ^c^
3						trace ^d^
4	CH_2_Cl (**1d**)		60 °C, 3 h	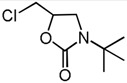	**4b**	79
5	CH_2_OPh (**1e**)		60 °C, 3 h	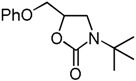	**4c**	90
6	CH_2_OMe (**1f**)		60 °C, 3 h	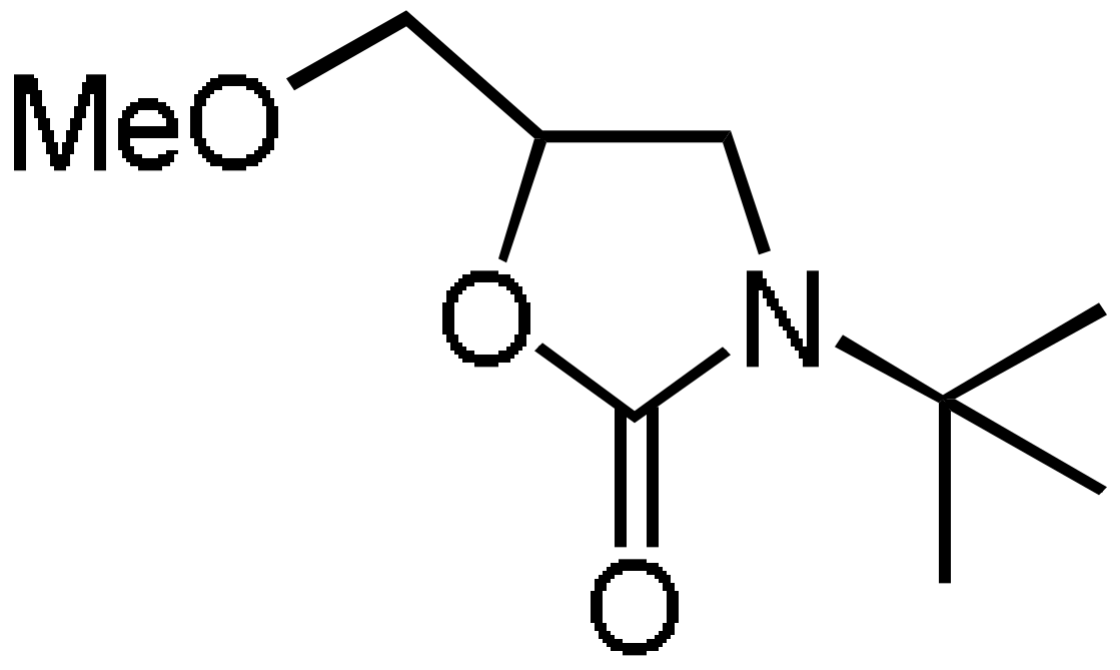	**4d**	99
7	Et (**1b**)	*n*-Bu (**3b**)	60 °C, 7 h	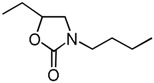	**4e**	49
8	Et (**1b**)	Ph (**3c**)	rt, 20 h	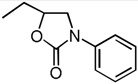	**4f**	64

^a^ InCl_3_ 0.25 mmol, Bu_2_SnI_2_ 0.5 mmol, epoxide **1** 5 mmol, isocyanate **3** 5.5 mmol, MeCN 1.5 mL, under nitrogen; ^b^ Determined by ^1^H-NMR; ^c^ Only Bu_2_SnI_2_ was used; ^d^ Only InCl_3_ was used.
